# αKLOTHO and sTGFβR2 treatment counteract the osteoarthritic phenotype developed in a rat model

**DOI:** 10.1007/s13238-019-00685-7

**Published:** 2020-01-16

**Authors:** Paloma Martinez-Redondo, Isabel Guillen-Guillen, Noah Davidsohn, Chao Wang, Javier Prieto, Masakazu Kurita, Fumiyuki Hatanaka, Cuiqing Zhong, Reyna Hernandez-Benitez, Tomoaki Hishida, Takashi Lezaki, Akihisa Sakamoto, Amy N. Nemeth, Yuriko Hishida, Concepcion Rodriguez Esteban, Kensaku Shojima, Ling Huang, Maxim Shokhirev, Estrella Nuñez-Delicado, Josep M. Campistol, Isabel Guillen-Vicente, Elena Rodriguez-Iñigo, Juan Manuel Lopez-Alcorocho, Marta Guillen-Vicente, George Church, Pradeep Reddy, Pedro Guillen-Garcia, Guang-Hui Liu, Juan Carlos Izpisua Belmonte

**Affiliations:** 1 Gene Expression Laboratory, Salk Institute for Biological Studies, 10010 North Torrey Pines Road, La Jolla, CA 92037 USA; 2 Wyss Institute for Biologically Inspired Engineering, Harvard University, Cambridge, MA 02115 USA; 3 Department of Genetics, Harvard Medical School, Boston, MA 02115 USA; 4 Integrative Genomics and Bioinformatics Core, Salk Institute for Biological Studies, 10010 North Torrey Pines Road, La Jolla, CA 92037 USA; 5 Universidad Católica San Antonio de Murcia (UCAM), Campus de los Jerónimos, No 135 12, 30107 Guadalupe, Spain; 6 Hospital Clinic of Barcelona, Career Villarroel, 170, 08036 Barcelona, Spain; 7 Department of Traumatology and Research Unit, Clinica CEMTRO, 28035 Madrid, Spain; 8 National Laboratory of Biomacromolecules, CAS Center for Excellence in Biomacromolecules, Institute of Biophysics, Chinese Academy of Sciences, Beijing, 100101 China; 9 University of Chinese Academy of Sciences, Beijing, 100049 China; 10 Institute for Stem cell and Regeneration, Chinese Academy of Sciences, Beijing, 100101 China; 11 Translational Medical Center for Stem Cell Therapy, Shanghai East Hospital, Tongji University School of Medicine, Shanghai, 200120 China

**Dear Editor,**

Homeostasis and repair are critical biological processes that allow for tissue and organ preservation and function in multi-cellular organisms. Their regulation and extension vary drastically across the animal kingdom, and mammals show limited tissue-specific regenerative capacity that declines with age. During aging, articular cartilage is one of the tissues that undergo substantial changes in the matrix structure, molecular composition, metabolic activity, and mechanical properties (Loeser et al. 2016). As a result, articular cartilage experiences impaired homeostasis and limited capacity to undergo repair, contributing to osteoarthritis (OA) development (Loeser et al. 2016). OA is the most prevalent musculoskeletal disorder among the elderly and is the leading cause of disability in the US due to pain associated with the disease (Zhang et al. 2016). Although symptomatic pain relief is possible (Zhang et al. 2016), treatments to cure the pathology are currently unavailable. Interestingly, contrary to the loss of homeostasis and repair capacity with age, during embryogenesis as well as a short period after birth, mammals seem to have a higher regeneration capacity (Vivien et al. 2016). These and other facts beg the question of whether therapeutic targets can be developed towards the enhancement of the low regenerative capacity observed during adulthood and worsen upon aging.

We thus focused our attention on two molecules, αKLOTHO and soluble Transforming growth factor-beta receptor 2 (sTGFβR2), that have been individually described in cartilage homeostasis. The inhibition of the transforming growth factor β isoform 1 (TGFβ1) appears to inhibit osteophyte formation despite increasing proteoglycans degradation (Scharstuhl et al. 2002), whereas αKLOTHO seems to act as an important inhibitor of extracellular matrix (ECM) degradation (Chuchana et al. 2018). Although TGFβ1 was considered as a reparative mediator by stimulating chondrocyte proliferation and inhibiting chondrocyte hypertrophy (Varela-Eirin et al. 2018), recent findings also provide substantial evidence about the contribution of TGF-β/Smad signaling in OA development and progression. Maintaining a balance in the TGFβ1 pathway appears to be key in regulating cartilage homeostasis, either the increase of activin receptor-like kinase (ALK) ALK1/ALK5 receptors ratio (Varela-Eirin et al. 2018) or a prolonged exposure to TGFβ1 have been demonstrated to boost chondrocyte hypertrophy (Bakker et al. 2001). In fact, the study of TGFβ1 levels in the knee joint of human patients suggests that active TGFβ levels are very low or absent in healthy articular joints, while drastically elevate in joint diseases such as OA (Scharstuhl et al. 2002). sTGFβR2, which lacks the membrane-binding domain and shows a higher affinity for TGFβ1 and β3 (De Crescenzo et al. 2003), can be used to modulate TGF-β pathway. The other molecule, αKLOTHO, was initially identified as an anti-aging molecule in mice and shown to be downregulated in the cartilage and synovial membrane upon aging and OA (Pásztói et al. 2009). Although its specific role in articular cartilage is still unknown, αKLOTHO seems to prevent apoptosis, oxidative stress, and immune reaction in other organs (Hu and Moe 2012), all pathways known to be involved in OA development. We then hypothesized that combining both the molecules could enhance the regenerative capacity to restore the articular cartilage structure and function after OA.

First, OA was chemically induced in rats by intra-articular injection of papain. This enzyme does not impact the chondrocytes; so, it would not impair the regeneration mechanism of the cartilage. We analyzed the rat knee joints four weeks after the papain injection by comparing the osteoarthritis control group (here on, OAC) and a healthy control group of rats (here on, HC) (Fig. S1). The Safranin-O staining of the OAC group showed diminished cartilage thickness with discontinued fibrillar surface and cellular clusters within the cartilage (Fig. S1A and S1B). Clear signs of early-stages of OA were found four weeks after papain treatment, according to the normalized Osteoarthritis Research Society International (OARSI) scores (see Supplementary Materials). The OAC group showed a clear grade 2 OA (Fig. S1C) as defined by the parameters analyzed. The OA grade in these samples was further supported by the increase in the number of cells undergoing apoptosis detected by tunel staining (Fig. S1D). Moreover, compared to the HC group, OAC group shows an increased area of expression of collagen type X (COL10A) and Runt-related transcription factor 2 (RUNX2) markers (Fig. S1E), as marked by the brackets in the figure. COL10A and RUNX2 expression in chondrocytes refer to the calcification of the ECM by the hypertrophy of the chondrocytes (Sacitharan 2019). They are regularly found in the deeper layer of the hyaline cartilage, where the bone is formed. Similarly, the presence of proteolytic enzyme matrix metalloproteinase 13 (MMP13) outside the chondrocytes within the matrix indicates cartilage damage and loss of joint function (Sacitharan 2019) (Fig. S1F). Additionally, the levels of chondrocyte markers, including, Sex determining region Y (SRY) Box 9 (SOX9), collagen type II (COL2A) and aggrecan (ACAN) were reduced in the OAC group when compared to HC, as shown by the immunostaining (Fig. S1G). Altogether these results demonstrate that four weeks of papain treatment recapitulated several cellular and structural OA phenotypes associated with the pathology in animals and humans. For instance, the loss of ECM homeostasis caused by proteoglycan-degrading enzymes such as the MMP13 is one of the main pathological features described in OA patients (Sacitharan 2019).

To test the combined effect of αKLOTHO and sTGFβR2 on OA progression and cartilage repair, both the soluble factors were included in adeno-associated virus (AAV) serotype DJ (AAV-DJ) particles to deliver into the knee joint by the intra-articular injection. AAV-DJ is a highly recombinogenic hybrid vector created from DNA shuffling of eight AAV serotypes (Grimm et al. 2008), including AAV2 and AAV5, which have been extensively used in rodent cartilage and arthritic joints (Kyostio-Moore et al. 2013). Moreover, AAV-DJ possesses a higher ability to evade immune neutralization than other serotypes and is a perfect candidate to efficiently deliver higher quantities of therapeutic DNA both *in vitro* and *in vivo* (Grimm et al. 2008). To examine the safety of the procedure, we first performed an intra-articular injection of AAV-DJ-Luciferase. The luciferase readout showed the AAV infection restricted to the knee joint without entering the bloodstream, avoiding the affection of other tissues (Fig. S2A). Next, the infection specificity of the AAV-DJ serotype was analyzed *in vitro* by using AAD-DJ designed to express a green fluorescent protein (GFP) (AAD-DJ-GFP) in synovial mesenchymal cells and chondrocytes. Although both the populations were transduced, a significantly higher efficiency was observed in synovial mesenchymal cells compared to chondrocytes (Fig. S2B and S2C). Similarly, injection of AAV-DJ-GFP *in vivo* into the knee demonstrated a low infection of SOX9+ cells (Fig. S2D). The efficacy of infection of mesenchymal stem cells rather than chondrocytes might be beneficial in limiting possible detrimental effects on the chondrocytes as a result of AAV infection. Also, the broad range of infection by the AAV-DJ within the joint would favor the presence of αKLOTHO and sTGFβR2 within the synovial fluid. Accordingly, high αKLOTHO and sTGFβR2 expression was confirmed in the synovial fluid of rats treated with AAV-DJ-αKLOTHO and -sTGFβR2 (here on, KT group) by ELISA and Western blot (WB) analysis (Fig. S2E and S2F). Specifically, the synovial fluid was obtained from rat knees injected with AAV-DJ-GFP or AAV-DJ-αKLOTHO and -sTGFβR2 after causing grade 2 OA (view Fig. S3C). These results demonstrate the effectivity and safety of the AAV-DJ intra-articular use.

In order to proceed with the *in vivo* experiments, we compared the effect of KLOTHO and sTGFbR2 individually or in combination using a new OA *in vitro* model using high TGFβ1 concentration (see Supplementary Materials). The results analyzed by qPCR showed that the combination of both soluble factors favors the inhibition of hypertrophic markers and ECM proteolytic enzymes when compared to the single factor treatments (Fig. S3A). Accordingly, also the chondrocytes treated with both factors, when combined, showed higher protein expression of ACAN than αKLOTHO or sTGFβR2 (Fig. S3B).

To test the effectiveness of αKLOTHO and sTGFβR2 in cartilage repair, rats treated with papain/cysteine were allowed to develop grade 2 OA before injecting AAV-DJ-GFP (SHAM) or AAV-DJ-αKLOTHO and -sTGFβR2 (KT). Then, the rats were then allowed to recover for 6-weeks to address the effect of the therapy (Fig. S3C). As expected, the SHAM group showed an even more significant deterioration of their cartilage six weeks after the viral injection, when compared to the OAC group. The Safranin-O, COL2A and ACAN staining showed not only an increased erosion and loss of the cartilage structure but also calcification of the matrix, as demonstrated by the drastic downregulation of the ECM components in the remaining fragments (Figs. 1A–D, S1A–C and S1G), shown by IHF, gene expression analysis, and WB. Furthermore, the immunohistological analysis showed a drastic decrease in the number of SOX9+ cells (Fig. 1D) and apoptotic cells (Fig. 1G). Also, the distribution pattern of COL10A and RUNX2 matched the OA phenotype, being found closer to the cartilage surface in the remaining fragments (Fig. 1I). The presence of MMP13 within the remaining ECM (Fig. 1H) co-relate with the reduced thickness of the cartilage in these animals (Fig. 1E). As a result, the OARSI score analysis classified the injury as grade 4 (Fig. 1F), indicating a clear progression into OA pathology.

**Figure 1:**
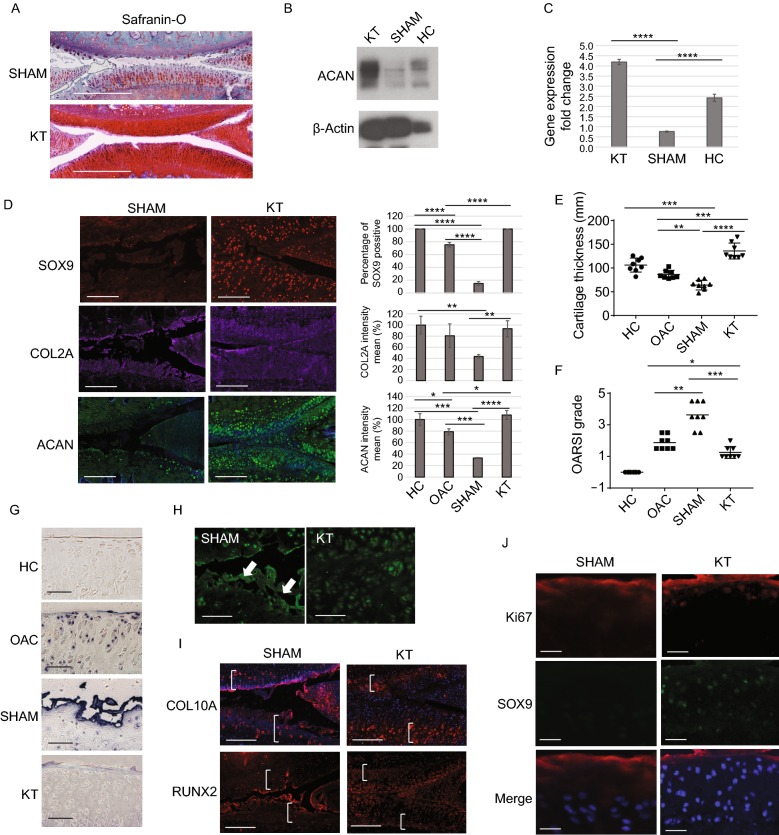
**sTGFβR2 and αKLOTHO intra-articular injection promotes ECM repair and avoids apoptosis**. (A) Representative Safranin-O images of knee joints (HC, *n* = 8; OAC, *n* = 8). Scale bars, 500 μm. (B) ACAN protein WB analysis. (C) *Acan* gene expression analyzed by qPCR. (D) Representative images from immunostaining detection of SOX9, COLl2A, and ACAN in knee sections, and their respective quantification. Quantification was performed within an area of 400 × 500 µm along the cartilage area. Quantification performed using Fiji software: HC, *n* = 3, COA, *n* = 3. Scale bars, 200 μm. Only ACAN images include DAPI co-staining (blue). (E) Quantification of the condyle cartilage thickness of HC and OAC rats (HC, *n* = 8; OAC; *n* = 8). The thickness was determined by measuring the condyle cartilage at three different positions throughout the cartilage area. Quantification performed using Fiji software. (F) Joint OA grade in rats based on the OARSI scoring system (HC, *n* = 8; OAC, *n* = 8). Data is expressed as means, and each data point represents an individual rat. (G) *In situ* cell death representative images (HC, *n* = 3; OAC). Blue colored cells represent apoptotic cells. Scale bars, 20 μm. (H) Representative images from immunostaining detection MMP13 in knee sections (HC, *n* = 3; OAC, *n* = 3). Scale bars, 200 μm. Staining outside the nuclear marked by arrows. (I) Representative images from immunostaining detection of COL10A and RUNX2 in knee sections (HC, *n* = 3; OAC, *n* = 3). Scale bars, 200 μm. Only COL10A images include DAPI co-staining (blue). Brackets indicate the cartilage area with COL10A or RUNX2 positive cells. (J) Representative images from immunostaining detection of Ki67 and SOX9+ cells. (SHAM, *n* = 3; KT, *n* = 3). Scale bars, 20 μm. Two-tailed *t*-test (unpaired) was used for statistical analysis of (C), (D), (E) and (F). *P < 0.05, **P < 0.01, ***P < 0.001, ****P < 0.0001. Error bars represent ± standard error (SEM)

On the other hand, the KT group showed a significantly improved phenotype 6-weeks after AAV injection. When compared to the OAC group, the Safranin-O staining showed the recovery of the cartilage structure and thickness in the KT group (Fig. 1A and 1E). Also, SOX9, COL2A and ACAN staining in the KT group further demonstrate the functional recovery of chondrocytes and the repair of the ECM components within the joint (Fig. 1D). Importantly, we observed a complete absence of apoptotic cells (Fig. 1G) and the appearance of proliferative cells marked by Ki67 staining (Fig. 1J) in the KT treated joints. Contrary to OAC and SHAM groups, in the KT treated joints, COL10A, and RUNX2 positive cells are mostly located in the lower levels as expected (Fig. 1I). This indicates that the injection of AAV-DJ-αKLOTHO and -sTGFβR2 inhibits the differentiation signals that lead to hypertrophy upon OA development. Also, we found the absence of MMP13 in the ECM of KT treated knees (Fig. 1H), which would help us explain the recovery of the matrix thickness upon the KT treatment (Fig. 1E). Based on all the improvements observed in the articular joints, the OARSI classification indicates that rats treated with αKLOTHO and sTGFβR2 recovered from a grade 2 OA to grade 1 OA within 6-weeks, while those treated with AAV-DJ-GFP progressed further to grade 4 (Fig. 1F). These results suggest that αKLOTHO and sTGFβR2 can improve the function of the cartilage tissue by restoring the SOX9+ cells and reducing the levels of a proteolytic enzyme, thereby reversing the OA phenotype.

To investigate the mechanisms behind αKLOTHO and sTGFβR2 effect, the cartilage tissues were isolated from all the groups for RNA-sequencing (RNA-seq) analysis. The RNA-seq analysis revealed upregulated and downregulated genes that were differentially expressed (DE) in the KT and HC groups when compared to the OAC and SHAM groups. Specifically, we found 136 common genes that were significantly up-regulated and 18 common genes significantly down-regulated in both KT versus SHAM and HC versus OAC comparisons (Fig. S4); and 217 unique genes that were significantly up-regulated and 118 unique genes significantly down-regulated in SHAM versus KT comparison that differ from HC versus OAC comparison (Fig. S5). Principal component analysis (PCA) of the RNA-seq data showed that KT and OAC samples were closer to the HC samples when compared to SHAM samples (Fig. 2A). Focusing on the major transcriptome alterations generated by the pathology, we reran the PCA with just the DE genes between HC and SHAM, which showed a closer distance between KT and HC when compared to both OAC and SHAM groups (Fig. 2B). Gene ontology (GO) analysis indicated that among the DE genes, those involved in the inflammatory response and immune response exhibited the most dramatic effect upon KT treatment (Fig. 2C and 2B).

**Figure 2:**
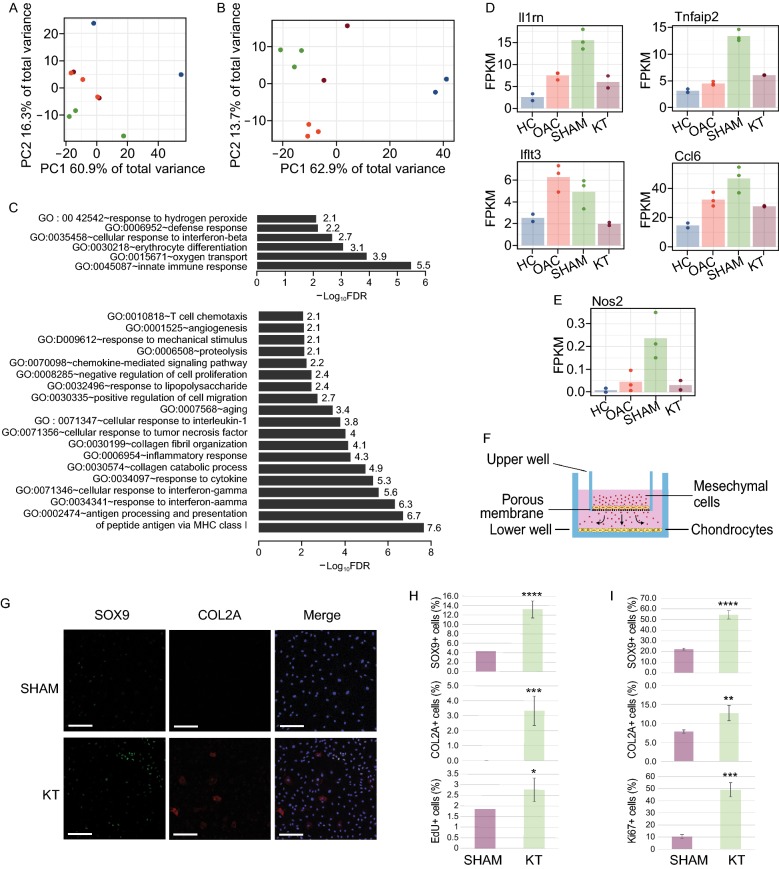
**sTGFβR2 and αKLOTHO inhibit OA-related immune response*in vivo*and help recover human chondrocyte markers**. (A) PCA on the top 500 most variable genes. Colors determine different conditions: HC (blue), OAC (red), SHAM (green), and KT (purple) (HC, *n* = 2; KT, *n* = 2; OAC, *n* = 3; and SHAM, *n* = 3). (B) PCA considering the DE genes between SHAM and HC. Colors determine different conditions: HC (blue), OAC (red), SHAM (green), and KT (purple) (HC, *n* = 2; KT, *n* = 2; OAC, *n* = 3; and SHAM, *n* = 3). (C) Barplots of the statistical enrichment scores from common DE genes between [KT vs. SHAM] and [HC vs. OAC] (upper barplot), and from KT vs. SHAM unique DE genes (not DE between HC vs. OAC) (lower barplot) according to GO enrichment analysis. (D) Gene expression plots of selected genes. (E) Gene expression plot of Nos2. (F) Schematic representation of the co-culture assay using human mesenchymal cells and chondrocytes (*n* = 3). Scale bars, 200 μm. (G) Representative immunostaining images of SOX9 and CoOL2A from chondrocytes used in co-culture experiments. Scale bars, 200 μm. (H) Immunostaining quantification of SOX9, COL2A and Ki67 in chondrocytes fused in co-culture experiments. (I) Immunostaining quantification of SOX9, COL2A and EdU in chondrocyte treated with sTGFβR2 and αKLOTHO recombinant proteins. In (A) and (B) Only Biological Process terms with FDR (false discovery rate) < 0.01 were shown in the plots. FDR values were shown in −log10 scale. In (D) and (E) Gene expression was normalized into FPKM values (Fragments/Kilobase/Million mapped reads) with the mean shown as the bar and each individual replicate shown as the dot. Colors determine different conditions: HC (blue), OAC (red), SHAM (green), and KT (purple) (HC, *n* = 2; KT, *n* = 2; OAC, *n* = 3; and SHAM, *n* = 3). Quantifications in (H) and (I) were performed by using Fiji software (SHAM, *n* = 3; KT, *n* = 3). Error bars represent ± standard error (SEM). Two-tailed *t*-test (unpaired) was used for statistical analysis. *P < 0.05, **P < 0.01, ***P < 0.001, ****P < 0.0001

Chondrocytes secrete proinflammatory cytokines under pathological conditions such as OA. Specifically, pro-inflammatory cytokines related to Nuclear factor-κB (*Nf-κB*) and Interleukin-1β (*Il-1β*) have been described to promote the action of MMPs contributing to the extracellular matrix degradation. Interestingly, when comparing OAC and SHAM groups to KT, our data showed downregulation of Interleukin-related genes such as Interleukin 1 receptor antagonist (*Il1rn*) (Figs. 2D and S5); Tumor necrosis factor (*Tnf*) -related/NF-κB-dependent genes such as *Tnf* alpha-induced protein 2 (*Tnfaip2*) (Figs. 2D and S5); interferon-related genes such as the Interferon-induced with tetratricopeptide repeats (*Ifit*) genes (Figs. 2D and S4); and cytokines or chemokines such as C-C motif chemokine ligand 6 (*Ccl6*) (Figs. 2D and S4) (Appleton et al. 2007). Therefore, the intra-articular injection of AAV expressing αKLOTHO and sTGFβR2 not only avoided the release of matrix-degrading enzymes to the ECM but also promoted the maintenance of the cartilage thickness. The role of αKLOTHO as an inhibitor of ECM degradation (Chuchana et al. 2018) supports and explains our results upon KT treatment. Moreover, our data demonstrate that αKLOTHO and sTGFβR2 together can successfully contribute to a complete ECM recovery after OA development. We speculate that sTGFβR2 contribution to the recovery of the anabolic-catabolic pathways balance could enhance the αKLOTHO ECM protective effect. The use of sTGFβR2 to sequester TGFβ1 may help to reduce the catabolic pathways while enhancing its anabolic effects (Bakker et al. 2001)

Our data also demonstrate that chondrocytes from the OAC group already show upregulation of proinflammatory cytokines and immune response-related factors as previously described for this pathology (Appleton et al. 2007). Interestingly, KT treatment not only downregulated the expression of some of those already expressed genes but also avoided the upregulation of other subsequent immune response factors. These results may be explained by the well-known role of TGFβ in inflammation during OA. TGFβ has been described to induce synovial lining cells to produce inflammatory factors, which can further stimulate hyaline chondrocytes hypertrophy (Bakker et al. 2001). Additionally, soluble αKLOTHO has also been reported to modulate the Phosphatidylinositol-4,5-bisphosphate 3-kinase/Protein kinase B (PI3K/AKT) and Wnt/β-catenin pathways, which are involved in cellular inflammatory responses (Hu and Moe 2012); and reduce cytokine levels implicated in other diseaseses (Hu and Moe 2012). Therefore, the cooperative activity of both factors may have helped to reduce the OA-related inflammatory response.

Moreover, our data also demonstrate how αKLOTHO and sTGFβR2 gene therapy can also avoid the subsequent destructive processes induced by the pro-inflammatory response by inhibiting the inducible nitric oxide synthase iNOS (*Nos2*) upregulation (Fig. S5). The KT treatment prevented the upregulation of *Nos2*, which was drastically increased in the SHAM group (Fig. 2E). During the inflammatory reaction, the nitric oxide (NO), generated by NOS2, has destructive effects leading to the chondrocyte death (Sacitharan 2019). Moreover, NO and the reactive oxygen species (ROS) appear to be the primary inducers of chondrocyte death during OA (Sacitharan 2019). Our data suggest that AAV-mediated αKLOTHO and sTGFβR2 expression avoided cartilage degradation by diminishing IL-1β-induced NO production by reducing *Il1rn* and *Nos2* mRNA levels in the chondrocytes. Although the anti-apoptotic role of αKLOTHO is not described in cartilage, it is well studied in other cell types such as (Hu and Moe 2012). This function of αKLOTHO could also sustain the downregulation of apoptosis demonstrated upon KT treatment.

Recent findings provide substantial evidence about the contribution of TGF-β/Smad signaling in the development and progression of OA (Bakker et al. 2001). Chondrocyte hypertrophy has been shown to be promoted by either an increase in ALK1/ALK5 receptors ratio during aging or longer exposure to TGFβ1 (Bakker et al. 2001; Varela-Eirin et al. 2018), indicating the importance of maintaining a balanced TGFβ pathway. Therefore, the high affinity of TGFβR2 receptor towards TGFβ1 and TGFβ3 (De Crescenzo et al. 2003) may be involved in the inhibition of chondrocytes hypertrophy as already indicated in other studies. This supports our observation of the downregulation of hypertrophic markers after KT treatment.

Currently, the most effective treatment for OA, besides arthroplasty, is autologous chondrocyte transplantation (Zhang et al. 2016). However, this treatment has several limitations, including the need to extract healthy donor cartilage by an additional surgical procedure, the limited expansion capacity of primary chondrocytes, and the difficulty of treating large-scale defects. Therefore, there is still the need to find effective therapies that can avoid surgical procedures and treat the pathology associated not only with aging but also with joint trauma.

In order to assess the effectiveness of KT treatment on human cartilage, we decided to test the effect of αKLOTHO and sTGFβR2 *in vitro* using human primary articular chondrocytes. The articular chondrocytic phenotype is characterized by the expression of cartilage-specific extracellular matrix components, predominantly COL2A and the cartilage-specific transcription factor SOX9 (Ma et al. 2013). However, the maintenance of differentiated phenotype *in vitro* is highly dependent on the culture condition, and one of the major obstacles accompanying the monolayer culture is the loss of hyaline chondrocyte phenotype, leading to chondrocyte dedifferentiation or hypertrophy (Ma et al. 2013).

To test the effect of αKLOTHO and sTGFβR2 on the phenotypic characteristics of the human hyaline chondrocytes in a monolayer culture condition, we first tried to mimic our *in vivo* model with the chondrocytes and mesenchymal cells that have a higher virus infection rate. For this purpose, we designed a co-culture experiment (see Supplementary Materials and Fig. 2F). Our results show that mesenchymal cells transduced with KT promoted the presence of a higher percentage of chondrocytes expressing the chondrocyte-specific markers SOX9 and COL2A, essential for maintaining the cellular identity and ECM formation, respectively (Fig. 2G and 2H). We also observed an increase in the number of cycling cells within the culture (Fig. 2H), which supports the effect of αKLOTHO on cell proliferation (Hu and Moe 2012) and suggests a possible mechanism involved in the cartilage re-growth after KT treatment.

Additionally, we also treated the human articular chondrocytes *in vitro* using recombinant αKLOTHO and sTGFβR2. The recombinant proteins also demonstrated a clear improvement in SOX9 and COL2A protein expression and cell proliferation (Fig. 2I).

Altogether these data on human cells highlight the possible applications of αKLOTHO and sTGFβR2 as potential factors for the maintenance of the chondrocytic phenotype in humans. We hypothesize that both factors could be useful to treat OA in humans, as our model recapitulates the OA phenotypes observed in human patients. However, additional studies will be needed to ensure its effectiveness and safety in the clinic.

Although a more detailed mechanism regarding how KT treatment improves cartilage repair still needs to be deciphered, the results reported here indicate that αKLOTHO and sTGFβR2 may, cooperatively, prevent OA progression by downregulating the immune response and promoting the joint tissue homeostasis.

## Electronic supplementary material

Below is the link to the electronic supplementary material.

Supplementary material 1 (PDF 551641 kb)
