# Supplementary material for: αKLOTHO and sTGFβR2 treatment counteract the osteoarthritic phenotype developed in a rat model
Source: Protein Cell. 2020 Jan 16;11(3):219–26. doi: 10.1007/s13238-019-00685-7 (PMC7026267; doi:10.1007/s13238-019-00685-7)
Supplement: Supplementary file 1 — Supplementary material 1 (PDF 551641 kb) [file 13238_2019_685_MOESM1_ESM.pdf]

SUPLEMENTARY METHODS

Cells isolation and culture

Human articular cartilage was obtained from de-identified healthy donors following informed consent for use in medical research, and rat articular cartilage was removed from the femoral and tibial condyles from a healthy rat under sterile conditions. Chondrocytes isolation and cultured was performed as described previously (Gosset et al. 2008).

Human mesenchymal cells were isolated from joint capsule connective tissues obtained from de-identified healthy donors following informed consent for use in medical research. Briefly, connective tissues were enzymatically digested as previously described (Yu et al. 2011) and subsequently, the stromal vascular fraction was isolated by centrifugation and maintained in basic culture medium.

AAV cloning and infection efficiency analysis

The AAV plasmids were constructed following standard cloning techniques. α-KLOTHO and sTGFβR2 were PCR amplified using primers as follows. α-KLOTHO-fwd-5'-cctgaa**cacctgcaacg**ggcctgccaccATGCTAGCCCGCGCCCCTCC α-KLOTHO-rev-cctgaacgtctc**gctagc**ttaTTACTTATAACTTCTCTGGCC sTGFβR2-fwd-5'-cctgaa**cacctgcaacg**ggcctgccaccAT GGGTCGGGGGTGCTCCGG-3' the uppercase is the overlap to the secretion signal of TGFβR2 receptor, the bold is the AarI recognition site that creates a NotI overhang. sTGFβR2-Rev-5'-ggcttgattgtgggccctctgggGTCGGGACTGCTGGTGGTGTATTC-3' the bold is the overlap to the extracellular domain of TGFβR2 receptor and the lower case matches the igg2a sequence used for overlap PCR. Igg2a-Fwd-5'-gaatacaccaccagcagtcccgacCCCAGAGGGCCCACAATCAAGCC-3' the bold is the

overlap to the mouse Igg2a FC region and the lower case matches the extracellular domain of TGFβR2 sequence used for overlap PCR. Igg2a-Rev 5'-cctgaa**cacctgcctta**ctagcTCATTTACCCGGAGTCCGGGAGAAG-3' the bold is the AarI recognition site that creates a NheI overhang.

AAVs were prepared using 293AAV cells (Cell Biolabs, Inc.) as described with minor modifications (Grieger et al. 2006). Briefly, cells were transfected using calcium phosphate and the virus purified by CsCl gradient. Virus titer was determined via qPCR using primers; ITR-F:5' GGAACCCCTAGTGATGGAGTT 3' and ITR-R: 5' CGGCCTCAGTGAGCGA 3'.

Eight male Sprague-Dawley rat were used to check the AAV-DJ-Klotho and AAV-DJ-sTGFβR2 infection efficiency. All of them were injected with papain/cystein to induce osteoarthritis and after 4 weeks, half of them were injected with AAV-DJ-GFP and the other half with AAV-DJ-αKLOTHO+AAV-DJ- sTGFβR2.

The level expression of αKLOTHO and sTGFβR2 were analyzed 2 weeks after the injection. Rats were euthanized by carbon dioxide and the synovial fluid of the experimental knee joints collected immediately afterward. First the skin was removed and 50μl of PBS was intra-articularly injected into the knee joint. The knee was bent to distribute the fluid throughout the joint and then the saline/synovial fluid was aspirated. Samples were centrifuged at 1000g for 20 min and the supernatant was stored at − 80 °C. The concentration of sTGFβR2 was tested by DYKDDDDK-Tag Detection ELISA KIT (Cat. No.501560 Cayman Chemical), while western blot analysis was used for sTGFβR2 and KLOTHO detection.

Animal experimental design

Twenty 250g Sprague Dawley (SD) female rats were divided into 4 groups of 8 animals per group: healthy control group (HC), osteoarthritis group control (OAC), AAV-DJ-αKLOTHO and sTGFβR2 treated group (KT) and a OA SHAM group (SHAM). OAC, KT and SHAM rats were treated with papain/cysteine according to methods.

OAC rats were sacrificed 4 weeks after the last papain/cysteine injection to determine the grade of OA reached at that point. The other 2 groups were subjected to intra-articular AAV treatment: AAV-DJ-GFP was injected to SHAM group, and AAV-DJ-αKLOTHO+AAV-DJ-sTGFβR2 to KT group. A total number of $2{,}5x10^{12}$ GC was injected per knee in 50μl of PBS. These two last groups were sacrificed six weeks after the virus injection. Both knee joints were harvested from each rat: one knee for histological analysis and the other for RNA isolation.

Osteoarthritis injury model

Induction of experimental osteoarthritis was performed by intra-articular injection of 100μl of 4% Papain (Sigma-Aldrich, P4762) prepared in PBS, followed by 50μl of 0.03M L-Cysteine (Sigma) also in PBS. The injections were performed three times (day 1, 4 and 7). This model promotes degradation of proteoglycans followed by disruption of cartilage micro-architecture and integrity of the knee joints (Pritzker 1994). All animal procedures were performed according to protocols approved by the IACUC and Animal Resources Department of the Salk Institute for Biological Studies.

RNA extraction

The cartilage surface was rinsed with saline and then dissected from the joint surfaces with a blade. Care was taken to avoid contamination by blood, bone, or synovium. The tissue was cut into small pieces, immersed in TRIzol (Ambion) and immediately snap-

frozen and stored at -80 ℃ until further use. Total RNA was isolated from cartilage tissue by using the TRIzol method. To determine the quality and integrity of total RNA samples (RIN), each RNA sample was run on a TapeStation automated electrophoresis analysis system (2200 TapeStation) according to the manufacturer's instructions. RNA concentrations were determined using Qubit Fluorometer 2.0.

Gene expression analysis

Total RNA was extracted from chondrocytes using the TRIzol reagent (Ambion) and kit RNA clean & Concentrator-5 with Zymo-spin IC Columns (Zymo Research Cat.No.R1016), and cDNA was synthesized using Maxima™ H Minus Reverse Transcriptase (ThermoFisher Scientific Cat.EP0751) following the manufacturer's instructions. Maxima SYBR Green mastermix was used to carry out qPCR. *βActin* was used as internal control. Primers sequences are the following: *Adamts4*, Forward 5'-CAAGGACTTCAATATTCCTCAG-3', Reverse 5'-GCAGTTCTTAGTGTTACAGG-3'; *Acan*, Forward 5'-AATGACTCTGGGATCTATCG-3', Reverse 5'-CAATGCCTTTTACTATGACCTC-3'; *MMP13*, Forward 5'-CGTGTGGAGTTATGATGATG-3', Reverse 5'-ATCTACTTTGTCGCCAATTC-3'; *Col10a1*, Forward 5'-TGCTGCCTCAAATACCCTTTC-3', Reverse 5'-TGGCGTATGGGATGAAGTATTG-3'; *Runx2*, Forward 5'-CCATAACGGTCTTCACAAATCC-3', Reverse 5'-TCTGTCTGTGCCTTCTTGGTTC-3'; *βActin*, Forward 5'-GGAGATTACTGCCCTGGCTCCTA-3', Reverse 5'-GACTCATCGTACTCCTGCTTGCTG-3'.

Histology and Immunofluorescence

The whole knee joint was prepared for histology as described in Kawamoto and Shimizu, 2000 (Kawamoto and Shimizu 2000). Samples were sectioned at a thickness of 7μm

using the method described by Kawamoto and Kawamoto, 2014 (Kawamoto and Kawamoto 2014). Sections were then stained using different methods. Safranin-O/Fast Green staining was performed according to standard procedures.

The thicknesses of the whole condylar cartilage was measured using the image analysis software Image J. The samples were evaluated by 2 blinded investigators and considering three different areas along the cartilage length.

For immunofluorescence analyses, sections were immunostained with antibodies and counterstained with 4,6-diamidino-2-phenylindole (DAPI). Imaging was performed using Slide Scanning Microscopy (Olympus VS-120 Virtual Slide Scanning Microscope).

The apoptosis detection was carried out on rat knee sections using *In situ* cell death detection AP kit (Roche) according to the manufacturer's protocol.

Western blot analysis

Samples were subjected to sodium dodecyl sulfate-polyacrylamide gel electrophoresis (SDS-PAGE), followed by transfer to polyvinylidine difluoride membranes (PVDF) and subsequent immunoblotting. After treatment of blocking buffer (TBS containing 0.1% Tween and 5% Skim milk) for 1 hr, the primary antibodies used were anti-αKlotho (1:1000), anti-Flag (1:1000) and anti-β-actin (1:2000) in blocking buffer for 16 hr, followed by incubation with HRP-conjugated anti-Rabbit IgG and HRP-conjugated anti-Mouse IgG in blocking buffer for 2 hr. HRP signal was detected with SuperSignalTM WestPico PLUS Chemiluminescent Substrate (Thermo Scientific, 34579).

Antibodies

Collagen II (NeoMarkers) at a 1:100 (v/v) dilution, Runx2 (Santa Cruz Biotechnology) at a 1:100 (v/v) dilution, MMP13 (Abcam) at a 1:100 (v/v) dilution, MMP3 (Abcam) at a 1:100 (v/v) dilution, Sox9 (Abcam) at a 1:100 (v/v) dilution, Collagen X (Abcam) at a

1:50 (v/v), Ki67 (BioLegend) at a 1:100 (v/v) dilution. Antigen retrieval was performed by heat mediation 5% hyaluronidase in an acetate buffer for 1 hour at 37 ℃. Immunoreactivity was visualized with a biotinylated anti-mouse IgG secondary antibody using the Avidin/Biotin Blocking Kit (Vector Laboratories, Inc) according to the manufacturer's protocols.

OARSI scoring system

The pathological changes of articular cartilage were graded using the Osteoarthritis Research Society International (OARSI) scoring System following the advanced methodology (Pritzker et al. 2006): A grade of 0 was received for normal and healthy cartilage; grade 1 was applied when the cartilage surface was intact but contained abrasion areas, hypertrophy and cellular clusters; grade 1.5 refers to a grade 1 that includes cell death; grade 2 cartilage presented discontinued fibrillar surface; grade 2.5 consist on a grade 2 that include the loss of matrix shown by less than 1/3 of Safranin-O staining; grade 3 was considered when fissures appeared up to the mid zone and Safranin-O stained less than 2/3 of the cartilage; grade 3.5 presented deeper fissures into the mid zone; grade 4 refers to matrix loss by delamination of the superficial zone; grade 4.5 showed excavation into the mid zone; grade 5 cartilage showed completely eroded unmineralized cartilage; grade 5.5 showed growth of hypertrophy cartilage after the erosion; and 6, the higher grade, indicates the more severe cartilage damage when deformation of the condyle appears.

RNA Sequencing and Data Analysis

Reads were mapped to the rn6 reference (Illumina iGenomes) by STAR [v2.5.1b (Dobin et al. 2013)] with default parameters. Only the uniquely mapped reads were used in the downstream analysis. Gene expression levels were calculated by summing reads that

were mapped across all exons of RefSeq genes by HOMER [v4.8 (Homer Software and Data Download)]. The differentially expressed (DE) genes were identified using DESeq2 [v1.18.1(Moderated estimation of fold change and dispersion for RNA-seq data with DESeq2) with the cutoff of logFC (log fold-change) > 0.5 and FDR (false discovery rate) < 0.05. Principle Component Analysis (PCA) was performed on the centered log-transformed FPKM values using R function prcomp.

Enrichment test was carried out by DAVID (v6.8) (Huang et al. 2009), in which case the genes of interest were used as input signals and the whole genome of rn6 was used as background. Only Biological Process terms with Benjamini-Hochberg FDR < 0.01 were used in this study. Data can be accessed through the GEO accession number GSE118559.

All the statistical analyses of RNA-Seq were performed in the R environment unless specifically mentioned [v3.4.3,(R: a language and environment for statistical computing)]. R packages ggplot2 (ggplot2) and gplots (Wickham et al. 2018) were used to plot the figures.

***In vitro* experiments**

Co-culture experiments: Corning® Transwell® polyester membrane cell culture inserts (Sigma) of 4μm pore size were used to evaluate the trans- *in vitro* effect of sTGFβR2 and αKLOTHO on human chondrocytes. Briefly, a p100 plate of human mesenchymal cells were transduced with $3x10^{12}$ of AAV-DJ-αKLOTHO and AAV-DJ-sTGFβR2. Two days after transduction, cells were reseed in the upper well of the co-culture chamber, and the same day, chondrocytes were seeded in the lower well of the chamber. Chambers of cells were incubated in 37 ℃ and 5% CO_2 conditions for 10 days.

Soluble factors experiment: Chondrocytes were plated at 60% confluence and treated with either BSA or 5ng/ml and 10ng/ml of αKLOTHO (Abcam ab84072) and sTGFβR2

(R&D Systems 241R2025) for 10 days. Cells were incubated in 37 ℃ and 5% CO_2 conditions and media with the factors or BSA were changed every 3 days.

OA *in vitro* model using TGFβ1: Protocol based on Chen et al. 2017. Briefly, rat chondrocytes were cultured at a density of $2x10^4$ cells/ml in growth medium and when the cells reached 75% confluence we started with the treatment. Serum-starved chondrocytes were treated with 10 ng/ml TGFβ1 (Peprotech cat#100-21B-10UG) alone for 24h, followed by co-treatment with Recombinant human αKLOTHO (Recombinant human Klotho, abcam ab84072) or Recombinant human TGFβR2 (Recombinant human TGFβRII protein, R&D 241-R2025) protein or both together. This experiments were designed to mimic the cellular events that occur in the condition of OA.

***In vivo* luciferase detection**

Six 300g Long Evans rats were used to test AAV-DJ intra-articular injection: AAV-DJ-Luc or AAV-DJ (empty vector, negative control). The luciferase was detected 2 weeks after injection using an IVIS Kinetic 2200 (Caliper Life sciences). 50 mg/kg D-Luciferin (Biosynth) was injected intra-articular and intraperitoneal. Imaging was captured 10 min after the D-luciferin injection.

Immunofluorescence of Cell Cultures

Previously to fixation, cells were incubated with EdU for 2h, according to standard protocols. Cells were then fixed using 4% PFA. Cells were permeabilized with 0.1% Triton X-100 in PBS for 20 min at room temperature (RT). After washing with PBS, cells were blocked using 1% BSA in PBS for 1 h and incubated with primary antibody at 4ºC overnight (Collagen II diluted 1:150 and Sox9 1:300). The secondary antibody was incubated 1h at RT. EdU staining was performed using Click-iT™ EdU Alexa Fluor™

488 Imaging Kit (Invitrogen). Counterstaining was performed using DAPI. Images were obtained using a Zeiss LSM 880 Rear Port Laser Scanning Confocal.

Flow cytometry analysis

GFP positive cells were detected using a FACS Canto II (BD Biosciences) after filtering (70 µm) and washing with 2% FBS/PBS.

Statistics

Quantification data is expressed as averages ± s.e. Statistical significance (*P values) was determined by an unpaired, two-tailed Student's *t*-test with Welch's correction (we do not assume an equal s.d. in each group). All analyses were performed with Prism 7 software from GraphPad (San Diego, CA, USA). Statistical significance was determined to be $P < 0.05$, **$P < 0.01$, ***$P < 0.001$, ****$P < 0.0001$.

Fig. S1 Recapitulation of early-stage OA phenotype in a rat model. (**A**) Representative Safranin-O images of knee joints (HC, n=8; OAC, *n*=8). Scale bars, 500µm. (**B**) Quantification of the condyle cartilage thickness of HC and OAC rats (HC, n=8; OAC; n=8). (**C**) Joint OA grade in rats based on the OARSI scoring system (HC, n=8; OAC, *n*=8). Data is expressed as means, and each data point represents an individual rat. (**D**) *In situ* cell death representative images (HC, n=3; OAC). Blue colored cells represent apoptotic cells. Scale bars, 20 µm. (**E**) Representative images from immunostaining detection of COL10A and RUNX2 in knee sections (HC, n=3; OAC, *n*=3). Scale bars, 200µm. Only COL10A images include DAPI co-staining (blue). Brackets indicate the cartilage area with COL10A or RUNX2 positive cells. (**F**) Representative images from immunostaining detection MMP13 in knee sections (HC, n=3; OAC, *n*=3). Scale bars, 200µm. Staining outside the nuclear marked by arrows. (**G**)

Representative images from immunostaining detection of SOX9, COL2A, and ACAN in knee sections, and their respective quantification. Quantification was performed within an area of 400x500 µm along the cartilage area. Quantification performed using Fiji software: HC, n=3, COA, n=3. Scale bars, 200µm. Only ACAN images include DAPI co-staining (blue). Two-tailed t-test (unpaired) was used for statistical analysis of (B), (C) and (G). **P* < 0.05, ***P* < 0.01, ****P* < 0.001, *****P* < 0.0001. Error bars represent ± standard error (SEM).

Fig. S2 sTGFβR2 and αKLOTHO were chosen to be delivered by AAV-DJ serotype. (**A**) Representative whole-body luminescence images on day 14 after AAV-DJ-Luciferase (LUC) or AAV-DJ-Empty (sham) intra-articular injection (LUC, *n*=3; Sham, *n*=3) (left) and quantification of luminescence (in arbitrary units, A.U.). (**B**) Flow cytometry analysis of AAV-DJ-GFP *in vitro* transduced rat synovial cells showed more than 15% transduction efficiency (upper, non-transduced cells; lower, AAV-DJ-GFP transduced cells) (n=3). (**C**) Flow cytometry analysis of AAV-DJ-GFP *in vitro* transduced rat chondrocytes showed less than 4% efficiency of transduction (upper, non-transduced cells; lower, AAV-DJ-GFP transduced cells) (n=3). (**D**) Representative images from immunostaining detection of AAV-DJ-GFP infected cells anti-GFP and anti-Sox9 antibodies. (SHAM, *n*=3; KT, *n*=3). Scale bars, 200µm. (**E**) ELISA quantification of sTGFβR2 (Flag-tagged) concentration in the rats synovial fluid after the treatment using the Flag tag (SHAM, n=4; KT, *n*=4). (**F**) Western blot analysis of αKLOTHO and sTGFβR2 shows a clear enrichment of both proteins in the synovial fluid (SHAM, n=4; KT, *n*=4).

Fig. S3 sTGFβR2 and αKLOTHO treatment downregulates the expression of hypertrophic markers and ECM proteolytic enzyme. (**A**) Bar plots represent the gene

expression levels of *Adamts4*, *Col10*, *Runx2* and *Mmp13* in rat chondrocytes treated with TGFβ1+K/T/KT *in vitro* (t0= before treatment; t24=24h with TGFβ1; 24+24K=24h with TGFβ1+24h with 24h with TGFβ1 and αKLOTHO; 24+24T=24h with TGFβ1+24h with 24h with TGFβ1 and sTGFβR2; 24+24KT=24h with TGFβ1+24h with 24h with TGFβ1, sTGFβR2 and αKLOTHO; 24+24C=24h with TGFβ1+24h with 24h with TGFβ1 alone). Data is expressed as mean and represent a minimum of two experiments. Two-tailed t-test (unpaired) was used for the statistical analysis. *$P < 0.05$, **$P < 0.01$, ***$P < 0.001$, ****$P < 0.0001$. Error bars represent ± standard error (SEM). (**B**) Western blot analysis of ACAN in rat chondrocytes treated with TGFβ1+K/T/KT *in vitro* using b-acting to normalize (1=t0; 2=t24; 3=24+24C; 4=24+24K; 5=24+24T; 6=24+24KT). (**C**) Schematic of the time course for the OA experiments. Female rats undergoing papain mediated OA were either sacrificed after 4week (OAC) or injected intra-articular with AAVDJ-GFP (SHAM) or AAV-DJ-sTGFβR2 and AAV-DJ-αKLOTHO (KT).

Fig. S4 sTGFβR2 and αKLOTHO treatment promote healthy gene expression patterns. Heatmap of gene expression for commonly up- and down- regulated DE genes between HC and KT that were differentially regulated in both OAC and SHAM groups. Colors indicated the gene-wise relative expression values across conditions (low=blue, high=red). Row dendrogram showed the hierarchical clustering result based on the similarity of gene expression profile.

Fig. S5 sTGFβR2 and αKLOTHO prevent the activation of immune response mechanisms related to OA. Heatmap of gene expression for common up- and down-regulated DE genes between SHAM and KT that differ from HC and KT. Colors indicated the gene-wise relative expression values across conditions (low=blue,

high=red). Row dendrogram showed the hierarchical clustering result based on the similarity of gene expression profile.

REFERENCES

Chen J-L, Zou C, Chen Y, et al (2017) TGFβ1 induces hypertrophic change and expression of angiogenic factors in human chondrocytes. Oncotarget 8:91316–91327.

Dobin A, Davis CA, Schlesinger F, et al (2013) STAR: ultrafast universal RNA-seq aligner. Bioinformatics 29:15–21.

Gosset M, Berenbaum F, Thirion S, Jacques C (2008) Primary culture and phenotyping of murine chondrocytes. Nat Protoc 3:1253–1260.

Grieger JC, Choi VW, Samulski RJ (2006) Production and characterization of adeno-associated viral vectors. Nat Protoc 1:1412–1428.

Huang DW, Sherman BT, Lempicki RA (2009) Bioinformatics enrichment tools: paths toward the comprehensive functional analysis of large gene lists. Nucleic Acids Res

Kawamoto T, Kawamoto K (2014) Preparation of thin frozen sections from nonfixed and undecalcified hard tissues using Kawamot's film method (2012). Methods Mol Biol

Kawamoto T, Shimizu M (2000) A method for preparing 2- to 50-μm-thick fresh-frozen sections of large samples and undecalcified hard tissues. Histochem Cell Biol

Pritzker KP (1994) Animal models for osteoarthritis: processes, problems and prospects. Ann Rheum Dis 53:406–420

Pritzker KPH, Gay S, Jimenez SA, et al (2006) Osteoarthritis cartilage histopathology: grading and staging. Osteoarthr Cartil 14:13–29.

Wickham H, Chang W, Henry L, et al (2018) ggplot2: Create Elegant Data Visualisations Using the Grammar of Graphics

Yu G, Wu X, Kilroy G, et al (2011) Isolation of murine adipose-derived stem cells. Methods Mol Biol 702:29–36.

Homer Software and Data Download. http://homer.ucsd.edu/homer/. Accessed 2 Aug 2018a

Moderated estimation of fold change and dispersion for RNA-seq data with DESeq2. https://www.ncbi.nlm.nih.gov/pmc/articles/PMC4302049/. Accessed 2 Aug 2018b

R: a language and environment for statistical computing. https://www.gbif.org/tool/81287/r-a-language-and-environment-for-statistical-computing. Accessed 2 Aug 2018c

ggplot2 - Elegant Graphics for Data Analysis | Hadley Wickham | Springer. https://www.springer.com/us/book/9780387981413. Accessed 2 Aug 2018d

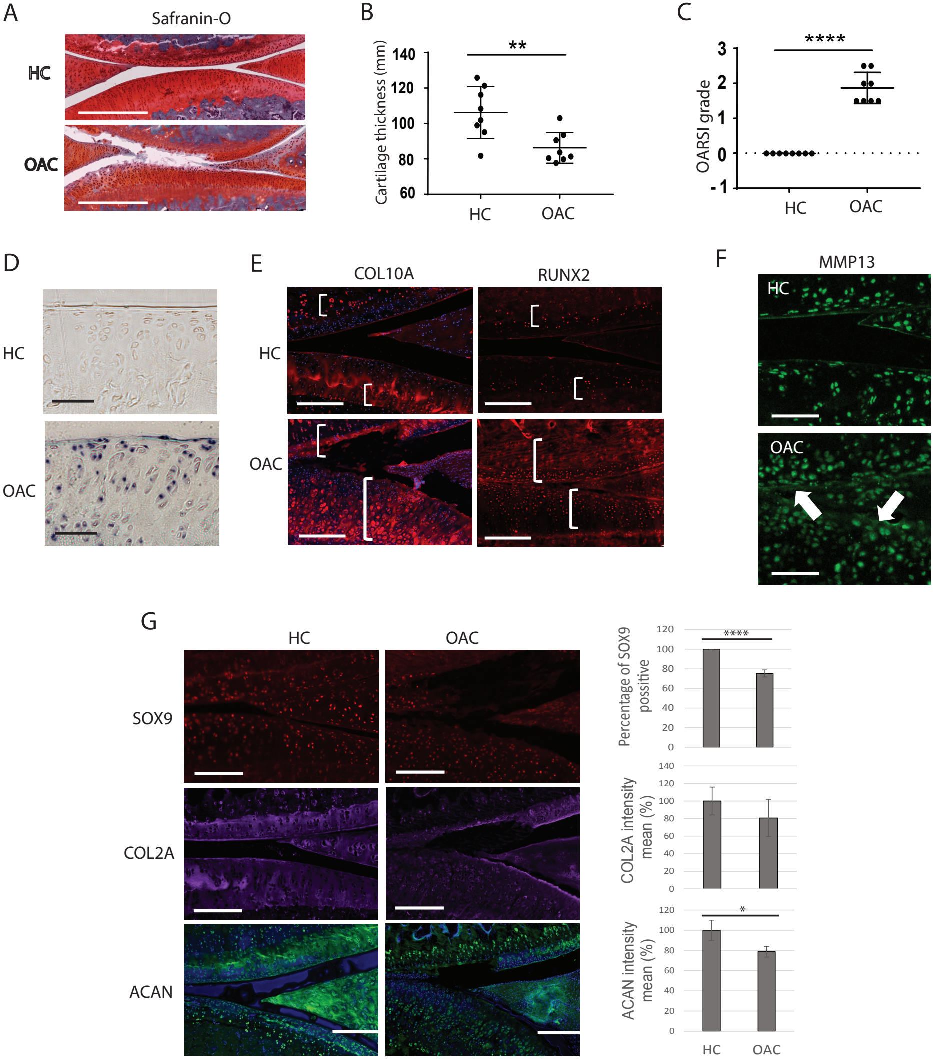
A
Safranin-O
HC
OAC
B
Cartilage thickness (mm)
**
140
120
100
80
60
HC
OAC
C
OARSI grade
****
3
2
1
0
-1
HC
OAC
D
HC
OAC
E
COL10A
RUNX2
HC
OAC
F
MMP13
HC
OAC
G
HC
OAC
SOX9
COL2A
ACAN
Percentage of SOX9 possitive
****
COL2A intensity mean (%)
ACAN intensity mean (%)
*
120
100
80
60
40
20
0
140
HC
OAC

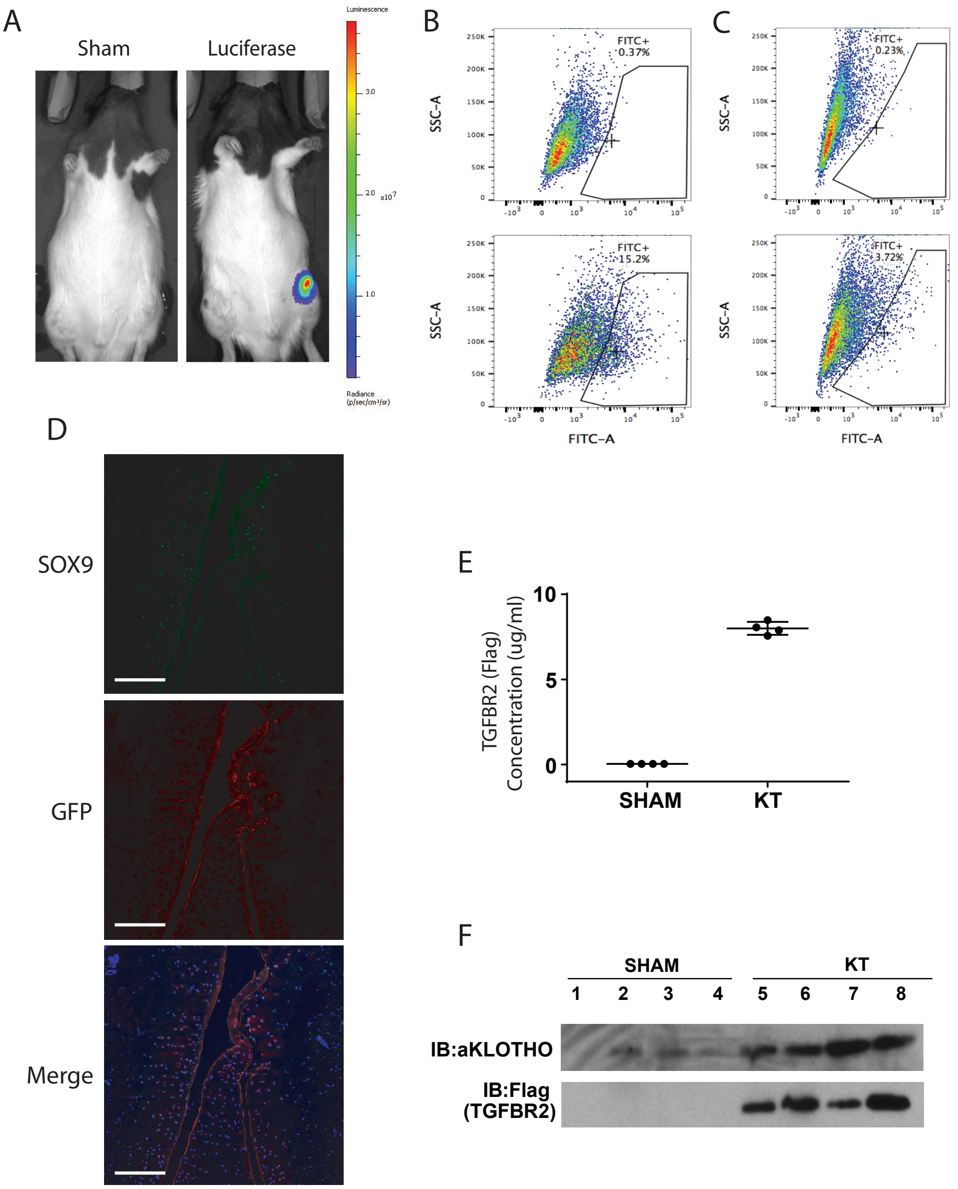
A
Sham
Luciferase
Luminescence
3.0
2.0
x10^7
1.0
Radiance
(p/sec/cm²/sr)
B
C
FITC+
0.37%
FITC+
0.23%
FITC+
15.2%
FITC+
3.72%
SSC-A
FITC-A
D
SOX9
GFP
Merge
E
TGFBR2 (Flag)
Concentration (ug/ml)
10
5
0
SHAM
KT
F
SHAM
KT
1
2
3
4
5
6
7
8
IB:aKLOTHO
IB:Flag
(TGFBR2)

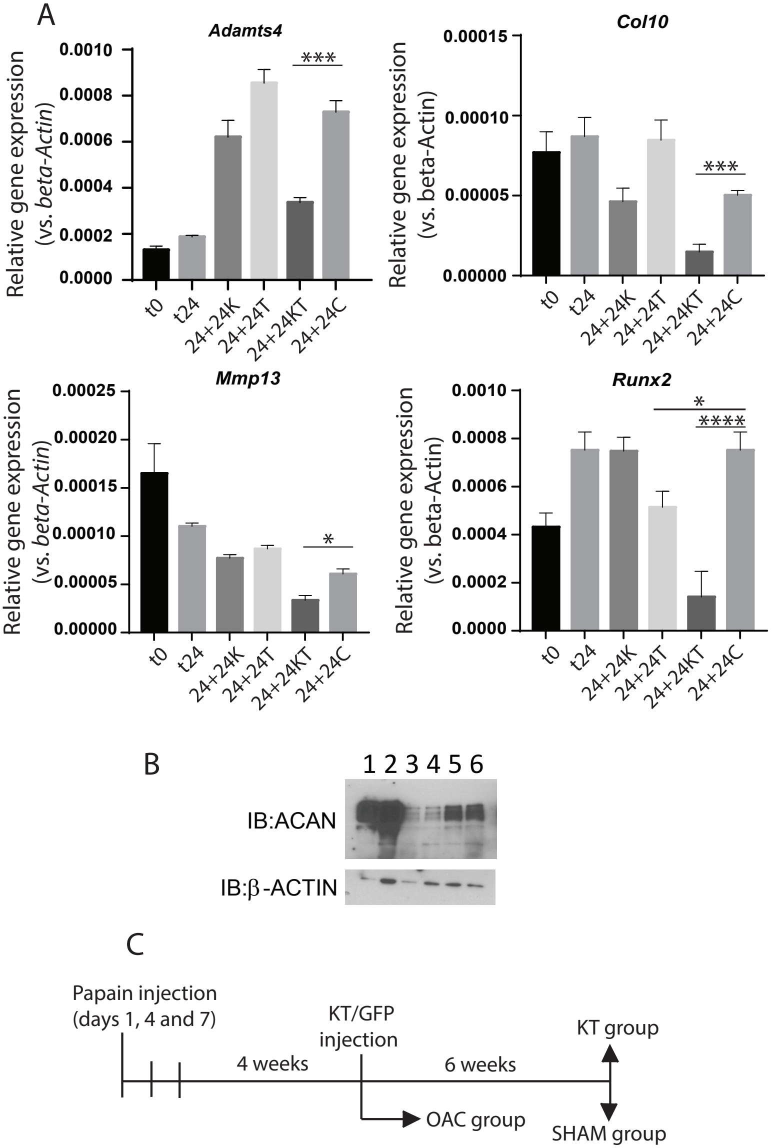

A
Adamts4
Relative gene expression (vs. beta-Actin)
0.0010
0.0008
0.0006
0.0004
0.0002
0.0000
***
t0
t24
24+24K
24+24T
24+24KT
24+24C
Col10
0.00015
0.00010
0.00005
0.00000
***
Mmp13
0.00025
0.00020
0.00015
0.00010
0.00005
0.00000
*
Runx2
*
****
B
1 2 3 4 5 6
IB:ACAN
IB:β-ACTIN
C
Papain injection (days 1, 4 and 7)
4 weeks
KT/GFP injection
6 weeks
KT group
OAC group
SHAM group

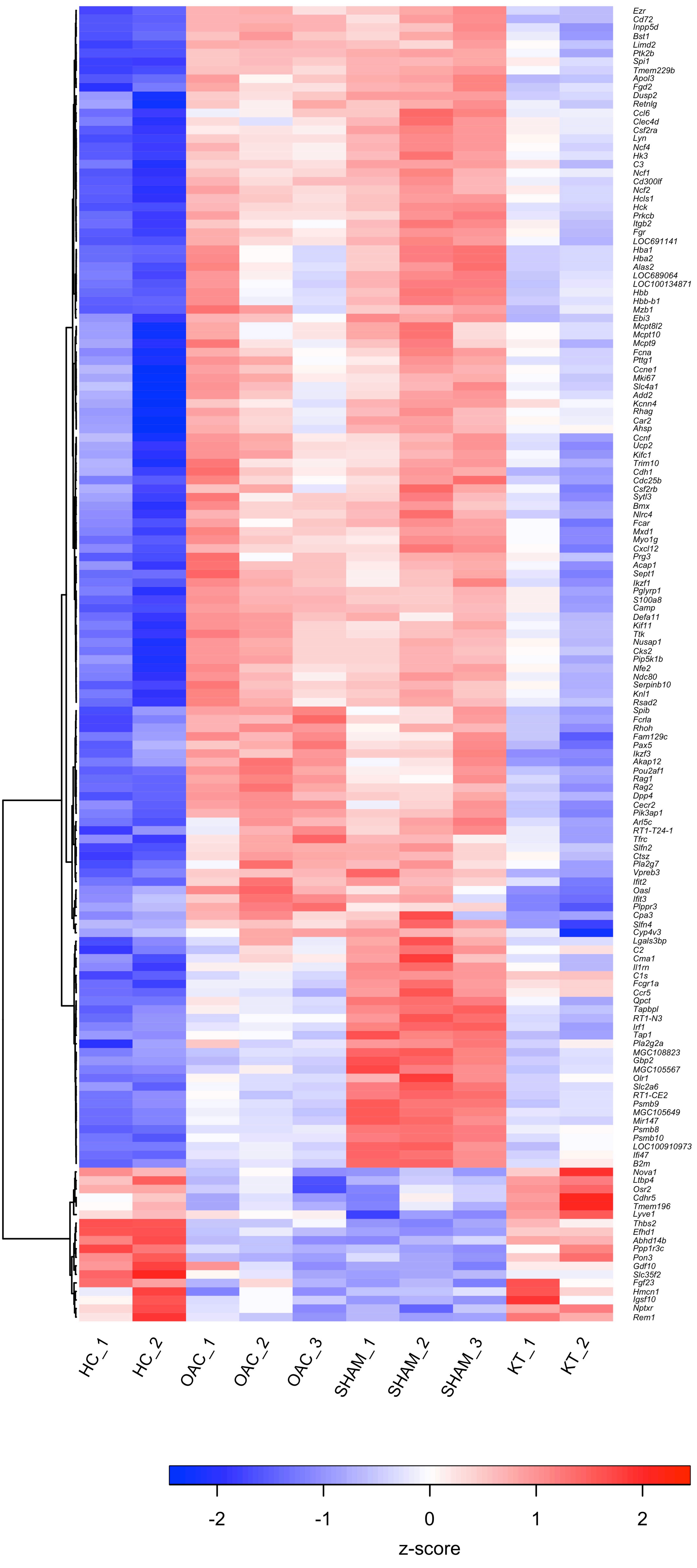
Ezr
Cd72
Inpp5d
Bst1
Limd2
Ptk2b
Spi1
Tmem229b
Apol3
Fgd2
Dusp2
Retnlg
Ccl6
Clec4d
Csf2ra
Lyn
Ncf4
Hk3
C3
Ncf1
Cd300lf
Ncf2
Hcls1
Hck
Prkcb
Itgb2
Fgr
LOC691141
Hba1
Hba2
Alas2
LOC689064
LOC100134871
Hbb
Hbb-b1
Mzb1
Ebi3
Mcpt8l2
Mcpt10
Mcpt9
Fcna
Pttg1
Ccne1
Mki67
Slc4a1
Add2
Kcnn4
Rhag
Car2
Ahsp
Ccnf
Ucp2
Kifc1
Trim10
Cdh1
Cdc25b
Csf2rb
Sytl3
Bmx
Nlrc4
Fcar
Mxd1
Myo1g
Cxcl12
Prg3
Acap1
Sept1
Ikzf1
Pglyrp1
S100a8
Camp
Defa11
Kif11
Ttk
Nusap1
Cks2
Pip5k1b
Nfe2
Ndc80
Serpinb10
Knl1
Rsad2
Spib
Fcrla
Rhoh
Fam129c
Pax5
Ikzf3
Akap12
Pou2af1
Rag1
Rag2
Dpp4
Cecr2
Pik3ap1
Arl5c
RT1-T24-1
Tfrc
Slfn2
Ctsz
Pla2g7
Vpreb3
Ifit2
Oasl
Ifit3
Plppr3
Cpa3
Slfn4
Cyp4v3
Lgals3bp
C2
Cma1
Il1rn
C1s
Fcgr1a
Ccr5
Qpct
Tapbpl
RT1-N3
Irf1
Tap1
Pla2g2a
MGC108823
Gbp2
MGC105567
Olr1
Slc2a6
RT1-CE2
Psmb9
MGC105649
Mir147
Psmb8
Psmb10
LOC100910973
Ifi47
B2m
Nova1
Ltbp4
Osr2
Cdhr5
Tmem196
Lyve1
Thbs2
Efhd1
Abhd14b
Ppp1r3c
Pon3
Gdf10
Slc35f2
Fgf23
Hmcn1
Igsf10
Nptxr
Rem1
HC_1
HC_2
OAC_1
OAC_2
OAC_3
SHAM_1
SHAM_2
SHAM_3
KT_1
KT_2
-2
-1
0
1
2
z-score

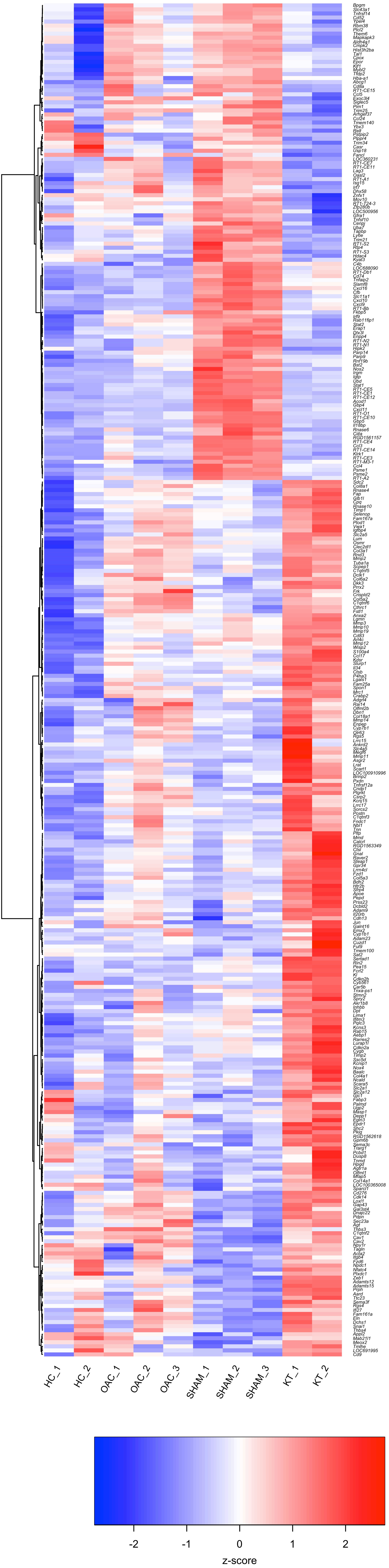

HC_1
HC_2
OAC_1
OAC_2
OAC_3
SHAM_1
SHAM_2
SHAM_3
KT_1
KT_2
-2
-1
0
1
2
z-score
